# 3-Acetyl-1-(2,6-dichloro­phen­yl)thio­urea

**DOI:** 10.1107/S160053681202925X

**Published:** 2012-07-04

**Authors:** Sharatha Kumar, Sabine Foro, B. Thimme Gowda

**Affiliations:** a Department of Chemistry, Mangalore University, Mangalagangotri 574 199, Mangalore, India; b Institute of Materials Science, Darmstadt University of Technology, Petersenstrasse 23, D-64287 Darmstadt, Germany

## Abstract

In the title compound, C_9_H_8_Cl_2_N_2_OS, the conformation of one of the N—H bonds is *anti* to the C=S group and the other is *anti* to the C=O group. Further, the conformations of the amide C=S and the C=O group are *anti* to each other. The 2,6-dichloro­phenyl ring and the 3-acetyl­thio­urea side chain are inclined to one another at a dihedral angle of 83.44 (5)°. An intra­molecular N—H⋯O hydrogen bond occurs. In the crystal, mol­ecules form inversion dimers through pairs of N—H⋯S hydrogen bonds.

## Related literature

For studies of the effects of substituents on the structures and other aspects of *N*-(ar­yl)-amides, see: Bhat & Gowda (2000 ▶); Gowda *et al.* (2003 ▶); Shahwar *et al.* (2012 ▶), of *N*-(ar­yl)-methane­sulfonamides, see: Gowda *et al.* (2007 ▶) and of *N*-chloro­aryl­sulfonamides, see: Gowda *et al.* (2005 ▶); Shetty & Gowda (2004 ▶).
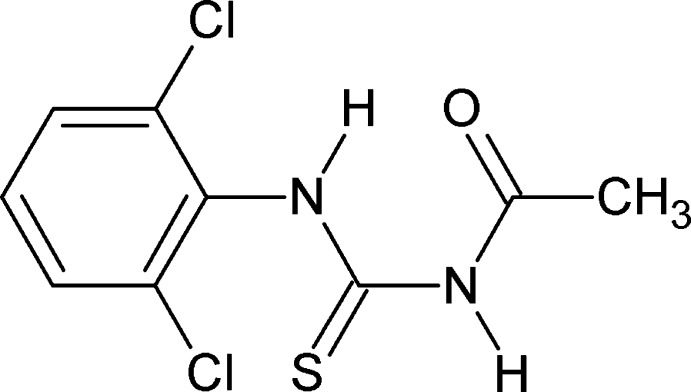


## Experimental

### 

#### Crystal data

C_9_H_8_Cl_2_N_2_OS
*M* *_r_* = 263.13
Triclinic, 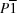

*a* = 7.729 (1) Å
*b* = 8.047 (1) Å
*c* = 10.015 (1) Å
α = 88.05 (1)°
β = 76.39 (1)°
γ = 66.57 (1)°
*V* = 554.24 (11) Å^3^
*Z* = 2
Mo *K*α radiation
μ = 0.75 mm^−1^
*T* = 293 K
0.44 × 0.44 × 0.04 mm

#### Data collection

Oxford Diffraction Xcalibur diffractometer with a Sapphire CCD detector
Absorption correction: multi-scan (*CrysAlis RED*; Oxford Diffraction, 2009 ▶) *T*_min_ = 0.735, *T*_max_ = 0.971
3638 measured reflections
2232 independent reflections
1930 reflections with *I* > 2σ(*I*)
*R*_int_ = 0.013

#### Refinement

*R*[*F* ^2^ > 2σ(*F* ^2^)] = 0.037
*wR*(*F* ^2^) = 0.095
*S* = 1.09
2232 reflections
143 parameters
3 restraints
H atoms treated by a mixture of independent and constrained refinement
Δρ_max_ = 0.30 e Å^−3^
Δρ_min_ = −0.37 e Å^−3^

### 

Data collection: *CrysAlis CCD* (Oxford Diffraction, 2009 ▶); cell refinement: *CrysAlis CCD*; data reduction: *CrysAlis RED* (Oxford Diffraction, 2009 ▶); program(s) used to solve structure: *SHELXS97* (Sheldrick, 2008 ▶); program(s) used to refine structure: *SHELXL97* (Sheldrick, 2008 ▶); molecular graphics: *PLATON* (Spek, 2009 ▶); software used to prepare material for publication: *SHELXL97*.

## Supplementary Material

Crystal structure: contains datablock(s) I, global. DOI: 10.1107/S160053681202925X/sj5247sup1.cif

Structure factors: contains datablock(s) I. DOI: 10.1107/S160053681202925X/sj5247Isup2.hkl

Supplementary material file. DOI: 10.1107/S160053681202925X/sj5247Isup3.cml

Additional supplementary materials:  crystallographic information; 3D view; checkCIF report

## Figures and Tables

**Table 1:** Hydrogen-bond geometry (Å, °)

*D*—H⋯*A*	*D*—H	H⋯*A*	*D*⋯*A*	*D*—H⋯*A*
N1—H1*N*⋯O1	0.84 (2)	1.94 (2)	2.631 (2)	139 (2)
N2—H2*N*⋯S1^i^	0.85 (2)	2.63 (2)	3.4252 (17)	158 (2)

Symmetry code: (i) 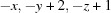.

**Fig. 1.**
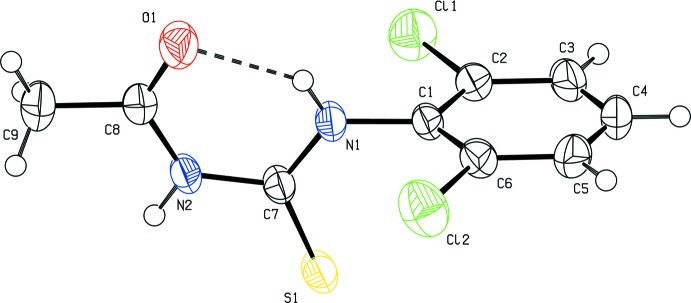
Molecular structure of the title compound, showing the atom labelling scheme and with displacement ellipsoids drawn at the 50% probability level. The intramolecular hydrogen bond is shown as a dashed line.

**Fig. 2.**
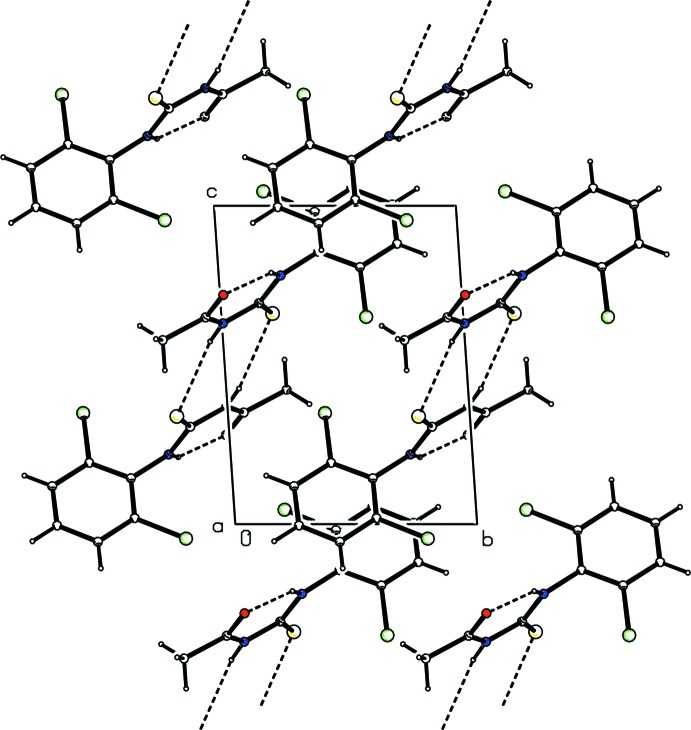
Molecular packing of the title compound with hydrogen bonding shown as dashed lines.

## supplementary crystallographic information

### Comment

Thiourea and its derivatives exhibit a wide variety of biological activities. As part of our studies of the substituent effects on the structures and other aspects of *N*-(aryl)-amides (Bhat & Gowda, 2000); Gowda *et al.*, 2003; Shahwar *et al.*, 2012); *N*-(aryl)-methanesulfonamides (Gowda *et al.*, 2007) and *N*-chloroarylsulfonamides (Gowda *et al.*, 2005; Shetty & Gowda, 2004), in the present work, the crystal structure of 3-acetyl-1-(2,6-dichlorophenyl)thiourea has been determined (Fig. 1).

The conformations of the amide C═S and the C═O are *anti* to each other, similar to the *anti* conformation observed in 3-acetyl-1-(2-methylphenyl)thiourea (Shahwar *et al.*, 2012). Further, the conformation of one of the N—H bonds is *anti* to the C═S and the other is *anti* to the C═O. The conformations of the two N—H bonds are are also *anti* to each other.

The side chain is tilted with respect to the 2,6-dichlorophenyl ring with torsion angles of C2—C1—N1—C7 = -86.22 (26)° and C6—C1—N1—C7 = 96.58 (24)°. The dihedral angle between the phenyl ring and the side chain is 83.44 (5)°.

The structure shows intramolecular hydrogen bonding between the NH hydrogen atom, attached to the 2,6-dichlorophenyl ring and the amide oxygen. In the crystal, the molecules form inversion type dimers through N—H···S intermolecular hydrogen bonds (Table 1, Fig.2).

### Experimental

3-Acetyl-1-(2,6-dichlorophenyl)-thiourea was synthesized by adding a solution of acetyl chloride (0.10 mol) in acetone (30 ml) dropwise to a suspension of ammonium thiocyanate (0.10 mol) in acetone (30 ml). The reaction mixture was refluxed for 30 min. After cooling to room temperature, a solution of 2,6-dichloroaniline (0.10 mol) in acetone (10 ml) was added and refluxed for 3 h. The reaction mixture was poured into acidified cold water. The precipitated title compound was recrystallized to constant melting point from acetonitrile. The purity of the compound was checked and characterized by its infrared spectrum.

Plate like dark yellow single crystals used in X-ray diffraction studies were grown in acetonitrile solution by slow evaporation of the solvent at room temperature.

### Refinement

H atoms bonded to C were positioned with idealized geometry using a riding model with the aromatic C—H = 0.93 Å, methyl C—H = 0.96 Å. The amino H atoms were freely refined with the N—H distances restrained to 0.86 (2) Å. All H atoms were refined with isotropic displacement parameters set at 1.2 *U*_eq_(C-aromatic, N) and 1.5 *U*_eq_ (*C*-methyl) of the parent atom.

### Crystal data

**Table:**

C_9_H_8_Cl_2_N_2_OS	*Z* = 2
*M**_r_* = 263.13	*F*(000) = 268
Triclinic, *P*1	*D*_x_ = 1.577 Mg m^−^^3^
Hall symbol: -P 1	Mo *K*α radiation, λ = 0.71073 Å
*a* = 7.729 (1) Å	Cell parameters from 2085 reflections
*b* = 8.047 (1) Å	θ = 2.8–27.7°
*c* = 10.015 (1) Å	µ = 0.75 mm^−^^1^
α = 88.05 (1)°	*T* = 293 K
β = 76.39 (1)°	Plate, dark yellow
γ = 66.57 (1)°	0.44 × 0.44 × 0.04 mm
*V* = 554.24 (11) Å^3^	

### Data collection

**Table:**

Oxford Diffraction Xcalibur diffractometer with a Sapphire CCD detector	2232 independent reflections
Radiation source: fine-focus sealed tube	1930 reflections with *I* > 2σ(*I*)
Graphite monochromator	*R*_int_ = 0.013
Rotation method data acquisition using ω and phi scans.	θ_max_ = 26.4°, θ_min_ = 2.8°
Absorption correction: multi-scan (*CrysAlis RED*; Oxford Diffraction, 2009)	*h* = −9→9
*T*_min_ = 0.735, *T*_max_ = 0.971	*k* = −10→9
3638 measured reflections	*l* = −7→12

### Refinement

**Table:**

Refinement on *F*^2^	Primary atom site location: structure-invariant direct methods
Least-squares matrix: full	Secondary atom site location: difference Fourier map
*R*[*F*^2^ > 2σ(*F*^2^)] = 0.037	Hydrogen site location: inferred from neighbouring sites
*wR*(*F*^2^) = 0.095	H atoms treated by a mixture of independent and constrained refinement
*S* = 1.09	*w* = 1/[σ^2^(*F*_o_^2^) + (0.0423*P*)^2^ + 0.2588*P*] where *P* = (*F*_o_^2^ + 2*F*_c_^2^)/3
2232 reflections	(Δ/σ)_max_ = 0.005
143 parameters	Δρ_max_ = 0.30 e Å^−^^3^
3 restraints	Δρ_min_ = −0.37 e Å^−^^3^

### Special details

**Table:**

Experimental. Absorption correction: CrysAlis RED (Oxford Diffraction, 2009) Empirical absorption correction using spherical harmonics, implemented in SCALE3 ABSPACK scaling algorithm.
Geometry. All e.s.d.'s (except the e.s.d. in the dihedral angle between two l.s. planes) are estimated using the full covariance matrix. The cell e.s.d.'s are taken into account individually in the estimation of e.s.d.'s in distances, angles and torsion angles; correlations between e.s.d.'s in cell parameters are only used when they are defined by crystal symmetry. An approximate (isotropic) treatment of cell e.s.d.'s is used for estimating e.s.d.'s involving l.s. planes.
Refinement. Refinement of *F*^2^ against ALL reflections. The weighted *R*-factor *wR* and goodness of fit *S* are based on *F*^2^, conventional *R*-factors *R* are based on *F*, with *F* set to zero for negative *F*^2^. The threshold expression of *F*^2^ > σ(*F*^2^) is used only for calculating *R*-factors(gt) *etc*. and is not relevant to the choice of reflections for refinement. *R*-factors based on *F*^2^ are statistically about twice as large as those based on *F*, and *R*- factors based on ALL data will be even larger.

### Fractional atomic coordinates and isotropic or equivalent isotropic displacement parameters (Å^2^)

**Table:**

	*x*	*y*	*z*	*U*_iso_*/*U*_eq_	
C1	0.0838 (3)	0.5813 (3)	0.14414 (19)	0.0322 (4)	
C2	0.2043 (3)	0.5857 (3)	0.0189 (2)	0.0359 (5)	
C3	0.3407 (3)	0.4272 (3)	−0.0553 (2)	0.0447 (6)	
H3	0.4218	0.4319	−0.1389	0.054*	
C4	0.3548 (3)	0.2629 (3)	−0.0042 (3)	0.0488 (6)	
H4	0.4466	0.1563	−0.0537	0.059*	
C5	0.2355 (4)	0.2534 (3)	0.1192 (3)	0.0465 (6)	
H5	0.2449	0.1416	0.1525	0.056*	
C6	0.1012 (3)	0.4131 (3)	0.1927 (2)	0.0374 (5)	
C7	−0.0252 (3)	0.8408 (3)	0.30587 (19)	0.0300 (4)	
C8	−0.3676 (3)	1.0659 (3)	0.3506 (2)	0.0356 (5)	
C9	−0.4980 (3)	1.2494 (3)	0.4221 (3)	0.0524 (6)	
H9A	−0.4576	1.3397	0.3769	0.079*	
H9B	−0.4901	1.2490	0.5164	0.079*	
H9C	−0.6296	1.2768	0.4187	0.079*	
N1	−0.0603 (2)	0.7439 (2)	0.21891 (17)	0.0342 (4)	
H1N	−0.175 (3)	0.782 (3)	0.210 (2)	0.041*	
N2	−0.1818 (2)	0.9960 (2)	0.36945 (17)	0.0349 (4)	
H2N	−0.156 (3)	1.053 (3)	0.426 (2)	0.042*	
O1	−0.4216 (2)	0.9862 (2)	0.28007 (18)	0.0530 (5)	
Cl1	0.18527 (10)	0.79226 (8)	−0.04530 (6)	0.05449 (19)	
Cl2	−0.04629 (11)	0.40151 (9)	0.34891 (6)	0.0607 (2)	
S1	0.19132 (8)	0.78798 (8)	0.33840 (6)	0.04599 (18)	

### Atomic displacement parameters (Å^2^)

**Table:**

	*U*^11^	*U*^22^	*U*^33^	*U*^12^	*U*^13^	*U*^23^
C1	0.0283 (9)	0.0309 (10)	0.0335 (10)	−0.0061 (8)	−0.0087 (8)	−0.0104 (8)
C2	0.0328 (10)	0.0362 (11)	0.0355 (10)	−0.0091 (9)	−0.0099 (8)	−0.0045 (8)
C3	0.0327 (11)	0.0510 (14)	0.0374 (11)	−0.0048 (10)	−0.0038 (9)	−0.0144 (10)
C4	0.0393 (12)	0.0385 (13)	0.0534 (14)	0.0040 (10)	−0.0152 (11)	−0.0198 (11)
C5	0.0501 (13)	0.0290 (11)	0.0562 (14)	−0.0061 (10)	−0.0214 (11)	−0.0052 (10)
C6	0.0375 (11)	0.0367 (11)	0.0378 (11)	−0.0129 (9)	−0.0110 (9)	−0.0062 (9)
C7	0.0291 (8)	0.0289 (10)	0.0276 (9)	−0.0089 (8)	−0.0025 (7)	−0.0048 (8)
C8	0.0324 (10)	0.0336 (11)	0.0340 (10)	−0.0077 (9)	−0.0045 (8)	−0.0046 (8)
C9	0.0411 (13)	0.0410 (13)	0.0574 (15)	0.0012 (10)	−0.0084 (11)	−0.0153 (11)
N1	0.0258 (8)	0.0321 (9)	0.0393 (9)	−0.0054 (7)	−0.0070 (7)	−0.0125 (7)
N2	0.0315 (9)	0.0324 (9)	0.0353 (9)	−0.0067 (7)	−0.0066 (7)	−0.0131 (7)
O1	0.0351 (8)	0.0514 (10)	0.0654 (11)	−0.0058 (7)	−0.0162 (8)	−0.0201 (8)
Cl1	0.0596 (4)	0.0476 (4)	0.0521 (4)	−0.0206 (3)	−0.0080 (3)	0.0075 (3)
Cl2	0.0744 (5)	0.0573 (4)	0.0485 (4)	−0.0325 (3)	−0.0004 (3)	0.0009 (3)
S1	0.0299 (3)	0.0539 (4)	0.0455 (3)	−0.0060 (2)	−0.0090 (2)	−0.0226 (3)

### Geometric parameters (Å, º)

**Table:**

C1—C2	1.384 (3)	C7—N1	1.329 (2)
C1—C6	1.388 (3)	C7—N2	1.385 (2)
C1—N1	1.424 (2)	C7—S1	1.664 (2)
C2—C3	1.384 (3)	C8—O1	1.211 (2)
C2—Cl1	1.725 (2)	C8—N2	1.376 (3)
C3—C4	1.374 (4)	C8—C9	1.503 (3)
C3—H3	0.9300	C9—H9A	0.9600
C4—C5	1.377 (4)	C9—H9B	0.9600
C4—H4	0.9300	C9—H9C	0.9600
C5—C6	1.384 (3)	N1—H1N	0.836 (16)
C5—H5	0.9300	N2—H2N	0.845 (16)
C6—Cl2	1.730 (2)		
			
C2—C1—C6	118.15 (18)	N1—C7—N2	115.94 (17)
C2—C1—N1	121.29 (19)	N1—C7—S1	124.10 (15)
C6—C1—N1	120.50 (18)	N2—C7—S1	119.95 (14)
C1—C2—C3	121.2 (2)	O1—C8—N2	122.41 (18)
C1—C2—Cl1	119.41 (15)	O1—C8—C9	122.4 (2)
C3—C2—Cl1	119.43 (18)	N2—C8—C9	115.21 (18)
C4—C3—C2	119.3 (2)	C8—C9—H9A	109.5
C4—C3—H3	120.4	C8—C9—H9B	109.5
C2—C3—H3	120.4	H9A—C9—H9B	109.5
C3—C4—C5	121.1 (2)	C8—C9—H9C	109.5
C3—C4—H4	119.4	H9A—C9—H9C	109.5
C5—C4—H4	119.4	H9B—C9—H9C	109.5
C4—C5—C6	118.8 (2)	C7—N1—C1	123.48 (17)
C4—C5—H5	120.6	C7—N1—H1N	116.3 (16)
C6—C5—H5	120.6	C1—N1—H1N	120.3 (16)
C5—C6—C1	121.4 (2)	C8—N2—C7	128.23 (17)
C5—C6—Cl2	118.92 (18)	C8—N2—H2N	117.7 (16)
C1—C6—Cl2	119.64 (15)	C7—N2—H2N	114.0 (16)
			
C6—C1—C2—C3	−1.0 (3)	N1—C1—C6—C5	177.63 (19)
N1—C1—C2—C3	−178.23 (18)	C2—C1—C6—Cl2	179.87 (15)
C6—C1—C2—Cl1	179.45 (15)	N1—C1—C6—Cl2	−2.8 (3)
N1—C1—C2—Cl1	2.2 (3)	N2—C7—N1—C1	179.64 (19)
C1—C2—C3—C4	0.7 (3)	S1—C7—N1—C1	0.7 (3)
Cl1—C2—C3—C4	−179.70 (17)	C2—C1—N1—C7	−86.2 (3)
C2—C3—C4—C5	0.2 (3)	C6—C1—N1—C7	96.6 (2)
C3—C4—C5—C6	−0.8 (3)	O1—C8—N2—C7	6.5 (4)
C4—C5—C6—C1	0.5 (3)	C9—C8—N2—C7	−172.7 (2)
C4—C5—C6—Cl2	−179.01 (17)	N1—C7—N2—C8	−2.4 (3)
C2—C1—C6—C5	0.3 (3)	S1—C7—N2—C8	176.56 (17)

### Hydrogen-bond geometry (Å, º)

**Table:**

*D*—H···*A*	*D*—H	H···*A*	*D*···*A*	*D*—H···*A*
N1—H1*N*···O1	0.84 (2)	1.94 (2)	2.631 (2)	139 (2)
N2—H2*N*···S1^i^	0.85 (2)	2.63 (2)	3.4252 (17)	158 (2)

Symmetry code: (i) −*x*, −*y*+2, −*z*+1.
